# Vital Statistics of Sheffield

**Published:** 1844-02

**Authors:** 


*Vital Statistics of Sheffield.*—One of the most prosperous trades in Sheffield is the silver plated manufacture. Until the last two years it has not shared the distress experienced by all engaged in making cutlery. The workmen belonging to this branch amount to 400, and form several unions, the restrictive laws of which are effective, even in times of bad trade. The earnings of men vary from 18 to 42 shillings, and of women from 8 to 15 shillings a week.

The workmen in this manufacture are sober, intelligent, and steady, and are, or were, in tolerable circumstances. This is attributable to various causes. First, the rule of the trades' union, which prohibits the masters from taking apprentices, has reduced the number of workmen, formerly superabundant. Secondly, the expensive materials and tools used in this trade, prevent that sudden conversion of men into masters which is so common in so many other occupations. Thirdly, children are rarely employed in this trade, much under fourteen years of age. Fourthly, the workmen are not liable to be thrown out of employment by machinery. This occupation is neither particularly detrimental to health nor conducive to longevity. In the Silversmith's Benefit Society, the mean age attained by those who die above the age of 40 is 59.12; while among the working classes, generally, of Manchester, Leeds, and Liverpool, it is 58-97; so that the advantage in favor of the silversmiths is slight indeed. In rural districts the mean age attained by persons above 40, is 68.76. What a difference!

Next come the *saw manufacturers.*

"The workmen in this branch of trade are, perhaps, in no degree inferior in intelligence, sobriety, and general good conduct, to those in the manufacture of which we have just treated. They have both, equally, their respective unions, which regulate wages, the introduction of apprentices, and which, in time of sickness, afford a weekly allowance."

In the branch called *par excellence* saw making, there are 208 journeymen, of whom about 20 are not in the union; 130 boys—being, indeed, more than the rules of the union allow; and about one woman or girl to every eight men.

Nine-tenths of the men are in sick clubs; nineteen out of twenty can read and write; and their average wages are 28 shillings a week, the extremes being 24 shillings and 45 shillings.

The *saw grinders* are fine healthy men, who generally live in the country, and often add the cultivation of a plot of ground, or even a small farm, to their mechanical occupation. They are peculiarly liable to accidents, and of 42 saw grinders who have died since 1821, 5 were killed by the breaking of stones.

Dr. Holland gives a list of 13 accidents, being a part of those which have happened to 78 living members in the union. Most of them are very serious, *e. g.* "3. Drawn over the stone; severe hurt; in bed nine months." "9. Arm severely lacerated; lame three months."

The *edge-tool makers* are well paid, but are a less intelligent class than those previously mentioned. The number of *foremen* and *strikers* is equal in this trade; the former are said to earn, on the average, £1. 14s., and the latter £1. 2s. a week.

The account is furnished by a manufacturer, and, though no doubt accurate as far as it goes, is defective in an important point. We do not learn from it how many months in the year the men are at work. The prices have been strictly enforced, even during the last three years, but for what portion of that time have the men obtained them?

Three-fourths of the workmen in this branch are in sick clubs; but only one among five adults can write. Trades that require strong arms rather than acute heads, will be favorable rather to sensuality than intelligence; a blacksmith drinks more than he reasons; and Dr. Holland finds that the forgers of Sheffield have big occiputs, but low, retreating foreheads.

The spring knife makers are badly paid, and badly educated. "A few superior workmen may earn from 30s. to 40s. per week. In the first manufactories of the town the average is from 16s. to 25s. But in many of the inferior manufactories, the workmen are receiving no more than 12s. or 16s.

Moreover, when Dr. Holland was writing, in June, 1843, this scale of wages was very greatly reduced.

This branch is not in union; not above half of the adults can read, and not one-fourth moderately well; about two-thirds only of the adults are in sick clubs.

Two circumstances combine to depress wages in this department. The most important is the smallness of the capital required to set up as a cutler. A few pounds are sufficient. The other one is the facility which this branch offers for the employment of children at an early age.

The *file trade*, as regards the circumstances of those employed in it, holds a middle place between the silversmiths and saw-makers, who are above, and the spring knife cutlers, who are below them.

The branch of the file trade called *forgers*, consists, like the edge-tool makers, of foremen and strikers; but the profits are more equally divided between the two classes of workmen in the present instance; a foreman averaging £1. 12s. 10d. a week, and a striker £1. 6s. 9d.

As to *file-cutters*, by reference to thirty books of file-cutters, men of *steady* habits, each having the assistance of a boy, the average per week was found to be £1. 18s. 6d. Eighty per cent. of the adults can read, and seventy can write. The proportion among the boys is rather greater; which may either show that education is making way, or that a certain number of adults forget their little scholastic learning.

The *fork-grinders* carry on the most unwholesome tıade in the kingdom. Fork-grinding is always performed on a dry stone, and the clouds of stony and metallic dust produced in the process, clog up the respiratory organs, and cause grinder's asthma, a disease which carries off its victims, in a majority of cases, before the age of thirty!

The better the state of a given trade in Sheffield, says Dr. Holland, the smaller is the proportion of minors employed in it. Thus in the silver-plated branch, the minors are to the adults as 16 to 100; and among the edge-tool makers, 12½ to 100. In the spring knife branch the per centage is about 25; but among the fork-grinders the minors actually out-number the adults, as there are 100 boys to only 97 men!

Of the ninety-seven, about thirty are at this moment suffering from grinder's asthma. It is remarkable that in this destructive trade the men are ill paid, but Dr. Holland does no say what their wages are. Nor is their education superior to their condition; for of the 197 men and boys, one hundred and nine can only read, and 69 can write.—*London Medical Gazette*, Sept. 8th, 1843.

